# Involvement of Sclera in Lattice Retinal Degeneration: An Optical Coherence Tomography Study

**DOI:** 10.3390/diagnostics14121295

**Published:** 2024-06-19

**Authors:** Dmitrii S. Maltsev, Alexey N. Kulikov, Maria A. Burnasheva, Alexander S. Vasiliev, Yana A. Kalinicheva, Alina A. Kazak

**Affiliations:** Department of Ophthalmology, Military Medical Academy, 21, Botkinskaya Str., 194044 St. Petersburg, Russia; alexey.kulikov@mail.ru (A.N.K.); maria.andreevna1@gmail.com (M.A.B.); shizolamp@gmail.com (A.S.V.); yanna_one@mail.ru (Y.A.K.); alinakazak96@gmail.com (A.A.K.)

**Keywords:** lattice retinal degeneration, snail-track degeneration, horseshoe retinal break, optical coherence tomography, rhegmatogenous retinal detachment

## Abstract

The aim of the study was to evaluate the local status of the sclera in lattice retinal degeneration. Patients with lattice degeneration, snail-track degeneration, or horseshoe retinal breaks were included. One lesion of a single eye in each patient was captured with cross-sectional optical coherence tomography (OCT) along and across the greatest lesion dimension. The maximum height of scleral indentation was measured and compared between different lesion types and between lattice lesions with and without retinal breakage or local detachment. The correlation between the maximum height of the scleral indentation of lattice lesions and the age of the patients was calculated. Seventy-five eyes of 75 patients (44.4 ± 14.7 years; 35 males and 30 females) were included. OCT showed variable local scleral indentation in 52 out of 55 (94.5%) lattice lesions, in five out of nine (55.5%) snail-tack lesions, and in three out of eleven (27.3%) horseshoe breaks. The maximum scleral indentation within lattice lesions, snail-tack lesions, and horseshoe breaks was 227.2 ± 111.3, 22.0 ± 49.2, and 88.5 ± 48.4 µm, respectively (*p* < 0.001 for snail-tack lesions and horseshoe breaks compared to lattice lesions). Lattice lesions with retinal breaks and/or local retinal detachment had statistically significantly lower scleral indentation than those without (*p* = 0.01). The height of the scleral indentation of lattice lesions was positively correlated with patient age (r = 0.51, *p* = 0.03). In conclusion, scleral indentation is one of the hallmarks of lattice retinal degeneration and may be associated with a reduced risk of rhegmatogenous retinal detachment.

## 1. Introduction

Lattice degeneration (LD) of the retina is one of the most common findings in the posterior segment of otherwise healthy eyes and is considered to be an important risk factor for rhegmatogenous retinal detachment [1,2]. Typically, lattice lesions typically appear at the retinal periphery as a variably pigmented elongated area of retinal thinning crossed by white retinal vessels with vitreous attachment at the borders of the lesion and vitreous liquefication above the lesion. A combination of retinal thinning and vitreous attachment results in an increased risk of retinal tear and consequent retinal detachment. Although LD is usually associated with myopia, it can also be found in hyperopic eyes, but rarely in high myopes [3]. Among various studies, the prevalence of LD in the healthy population varies from around 2% to 5% [4]. 

The presence of LD may indicate the need for laser prophylaxis. However, the relatively low incidence rate of retinal detachment (around 0.02%) suggests that only a small number of the lesions provide a clinically meaningful risk [5,6]. Consequently, only symptomatic eyes are treated with lasers on a routine basis. On the other hand, the prevalence of lattice lesions among eyes with retinal detachment can be as high as 30% [1].

Despite being well-known and widely distributed in the population, the etiology of LD is poorly understood. It has been variously suggested that lattice lesions may result from local vitreous traction, primary choroidopathy, local retinal ischemia, or defects of the inner limiting membrane [1]. However, none of these hypotheses completely fit the existing histopathology and clinical data. 

Recent advances in multimodal imaging have provided new opportunities for diagnosing LD and have also revealed the variable phenotypes of lesions [7]. Two technologies, infrared scanning laser ophthalmoscopy and optical coherence tomography, suggest involvement of the sclera, at least in some lattice lesions. Particularly, scleral involvement has appeared on cross-sectional optical coherence tomography (OCT) as a scleral indentation in the middle of the lesion [8]. However, the frequency, detailed characteristics, and clinical meaning of scleral involvement in LD remain unknown.

The aim of this study was to evaluate the local status of the sclera in lattice lesions and to elucidate its clinical significance using OCT.

## 2. Materials and Methods

This was a single-center prospective study that included patients with peripheral retinal abnormalities, including LD, snail-track lesions, and horseshoe retinal breaks. The study cohort was recruited from candidates for LASIK surgery, individuals passing their routine ophthalmic examination, and patients with symptomatic retinal breaks. Medical records and imaging data were analyzed for patients who did not meet exclusion criteria, including significant optical media opacities, an inability to capture the entire lesion, myopia greater than 6.0 D, an undetermined type of the lesion, and other retinal comorbidities including diabetic retinopathy, age-related macular degeneration, or retinal vein occlusions. Only one eye of every patient and one lesion from one eye were included.

A lattice degeneration lesion was defined as an elongated, typically concentric or perivascular peripheral lesions with white or sheathed vessels and pigmentary clusters or atrophy presented in different combinations. Snail-track degeneration was defined as an elongated, concentric, non-pigmented lesion with sharp borders and multiple glinting flecks. 

All patients received a standard ophthalmic examination, including indirect ophthalmoscopy, by a single experienced retina specialist (DM). When the lesion type was confirmed, the patient received an OCT examination with SOLIX OCT (Optovue, Fremont, CA, USA), capturing the lesion area. The SOLIX OCT device operates with a light source of 840 nm at 120 kHz scanning speed and uses Full Range technology to obtain an 18 mm cross-sectional scan capturing peripheral lesions. 

In order to perform peripheral OCT imaging, the patient was seated in front of the OCT device as normal and instructed to look in the direction of the lesion. Some head tilt in the direction of the lesion was applied if needed. Auto adjustment was performed, and the scan was positioned on the lesion region according to the display in the infrared fundus image. A scan was then taken along and across the greatest lesion dimension. In the majority of cases, this approach allowed us to capture the lesion with sufficient quality and with a reliable correlation to the scan location. Additionally, for a detailed evaluation of the shape of the scleral indentation, an 8 mm radial scan consisting of twelve cross-sectional scans was captured in the middle of the lesion.

**Figure 1:**
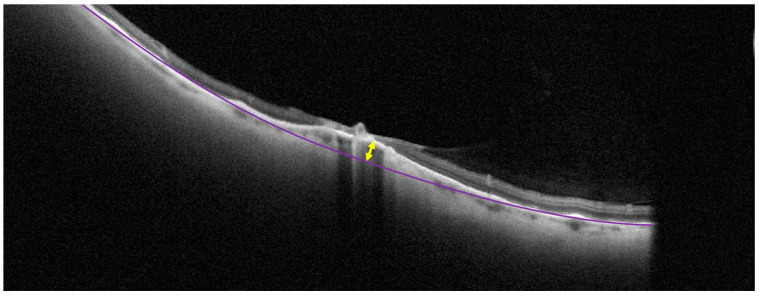
Measurement of choroidal indentation on a cross-sectional optical coherence tomography scan. The purple line represents the level of retinal pigment epithelium. The yellow arrow shows the maximum scleral indentation in the middle of the lesion.

The height of the scleral indentation was measured using scans of the lesion across and along its greatest dimension. The measurements were performed in ImageJ 1.53k (NIH, Bethesda, CA, USA). To measure the height of the scleral indentation, first the line of the retinal pigment epithelium (RPE) was reconstructed based on the RPE line along with the lesion area using an ellipsoid curve. Next, the distance between the highest point of the actual RPE line and the reconstructed line beneath it was measured (Figure 1). 

**Figure 2:**
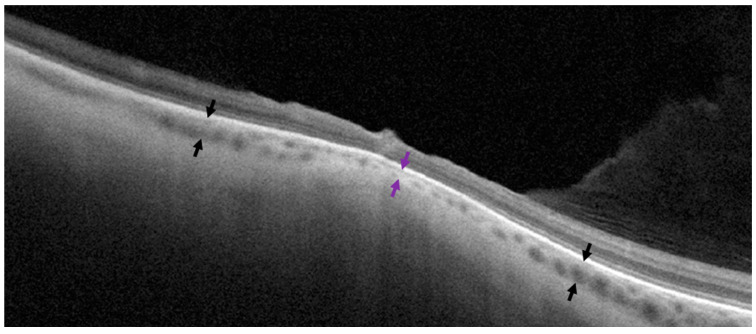
Measurement of choroidal thickness on a cross-sectional optical coherence tomography scan. Purple arrows show choroidal thickness in the middle of the lesion. Black arrows represent choroidal thickness in unaffected neighboring regions on both sides of the lesion.

Additionally, choroidal thickness was measured within the highest point of the lesion and at an adjacent normal region on the same scan passing across the lesion’s greatest dimension. Choroidal thickness was defined as the mean of three consecutive measurements of the distance between the lower border of the RPE and the choroidoscleral junction. The choroidal thickness in unaffected regions was calculated as the mean value of measurements taken from both sides of the lesion (Figure 2). Choroidal thickness at the lesion area was expressed as a percentage of the choroidal thickness of unaffected regions. For other types of lesions, the measurements were performed in the same manner. The presence of subretinal fluid and/or retinal breaks was defined based on ophthalmoscopic and OCT examinations.

Statistical analysis was performed using MedCalc 18.4.1 (MedCalc Software, Ostend, Belgium). The height of scleral indentation was compared between different types of lesions and different scan positions (passing along and across the greatest lesion dimension) using one-way ANOVA. The Bonferroni post hoc test was applied to correct multiple comparison errors. The correlation between indentation height and patient age was calculated using the Spearman rank correlation coefficient; *p* < 0.05 was considered statistically significant.

## 3. Results

Seventy-five eyes of 75 patients (44.4 ± 14.7 years; 35 males and 30 females) were included (Table 1).

OCT showed the presence of variable local scleral indentation in 52 out of 55 (94.5%) lattice lesions, in five out of nine (55.5%) snail-tack lesions, and in three out of eleven (27.3%) horseshoe breaks. The prevalence of scleral indentation was statistically significantly higher in LD compared to both snail-track degeneration and horseshoe break (*p* < 0.001). Eighteen out of 55 lattice lesions had retinal breaks, including ten with local subretinal fluid.

**Figure 3:**
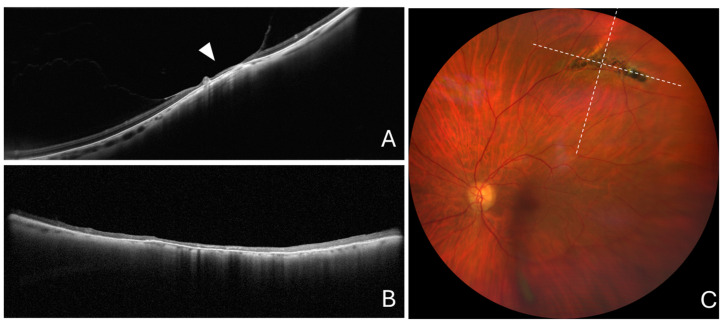
Cross-sectional optical coherence tomography and color fundus photography in a typical lattice lesion without retinal breaks. (**A**) Cross-sectional optical coherence tomography scan passing across the greatest lesion dimension showing notable scleral indentation (arrowhead). (**B**) Cross-sectional optical coherence tomography scan passing along the greatest lesion dimension, showing no scleral indentation. (**C**) Color fundus photography showing a typical lattice lesion. The dashed lines show the position of optical coherence tomography scans.

The mean scleral indentation across and along the lattice lesion was 227.2 ± 111.3 and 7.0 ± 23.8 µm, respectively (*p* < 0.001) (Figure 3). The choroidal thickness within the lattice lesion and in the unaffected neighboring region was 63.5 ± 38.4 and 135.6 ± 44.5 µm, respectively. The mean percentage choroidal thickness within the lattice lesion was 49.0 ± 24.6% (Table 1). Evaluation of radial OCT patterns revealed maximum scleral indentation on the scans passing across the greatest lesion dimension and no indentation on the scans passing along the greatest lesion dimension.

**Figure 4:**
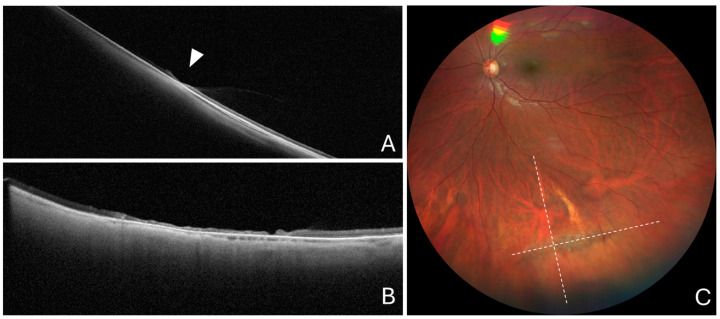
Cross-sectional optical coherence tomography and color fundus photography in a snail-track lesion. (**A**) Cross-sectional optical coherence tomography scan passing across the greatest lesion dimension showing local choroidal thinning with no scleral indentation (arrowhead). (**B**) Cross-sectional optical coherence tomography scan passing along the greatest lesion dimension, showing no scleral indentation. (**C**) Color fundus photography showing a typical snail-track lesion. Dashed lines show the position of optical coherence tomography scans.

The mean scleral indentation on the scan passing across the snail-track lesion was 36.8 ± 51.2 and 0.0 µm on the orthogonal OCT scan. The choroidal thickness in the middle of the lesion and in the unaffected neighboring region was 45.1 ± 16.1 and 117.9 ± 26.7 µm, respectively. The mean percentage choroidal thickness within the lesion was 40.2 ± 16.7% (Figure 4).

**Figure 5:**
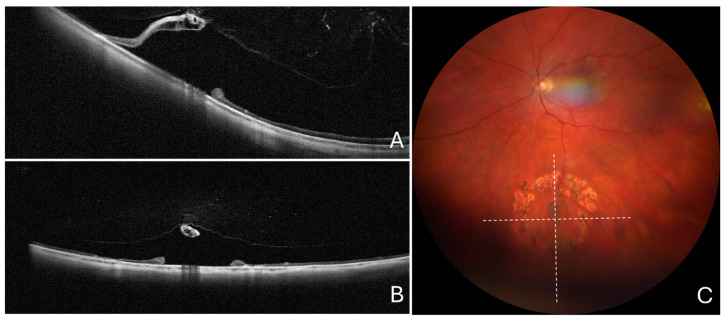
Cross-sectional optical coherence tomography and color fundus photography in a horseshoe retinal break. (**A**) Cross-sectional optical coherence tomography scan passing along the retinal flap showing no scleral indentation or choroidal thinning. (**B**) Cross-sectional optical coherence tomography scan passing across the retinal flap showing no scleral indentation. (**C**) Color fundus photography showing a horseshoe retinal break surrounded by post-laser scars. The dashed lines show the position of optical coherence tomography scans.

The maximum scleral indentation within the horseshoe break was 22.0 ± 49.2 and 0.0 µm on the orthogonal OCT scan. The choroidal thickness within the horseshoe break in the middle of the lesion and in the unaffected neighboring region was 138.5 ± 18.4 and 140.6 ± 14.5 µm, respectively. The mean percentage choroidal thickness within the horseshoe break was 99.6 ± 0.03% (Figure 5).

**Figure 6:**
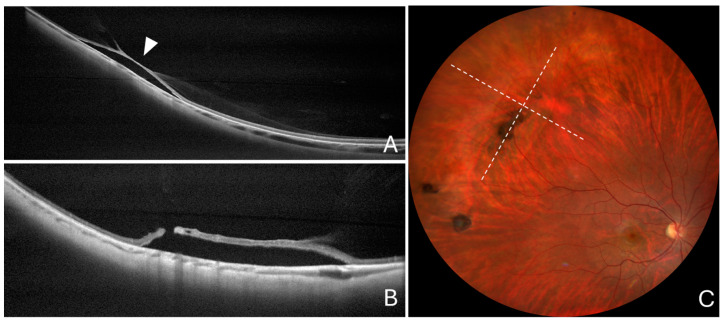
Cross-sectional optical coherence tomography and color fundus photography in a lattice lesion with retinal breaks and local retinal detachment. (**A**) Cross-sectional optical coherence tomography scan passing across the greatest lesion dimension, showing no scleral indentation and mild choroidal thinning (arrowhead). (**B**) Cross-sectional optical coherence tomography scan passing along the greatest lesion dimension, showing no scleral indentation. (**C**) Color fundus photography showing a lattice lesion and local retinal detachment. The dashed lines show the position of optical coherence tomography scans.

Lattice lesions with local retinal detachment and/or retinal breaks had statistically significantly lower scleral indentation than those without, 244.4 ± 87.9 and 141.2 ± 133.0 µm, respectively (*p* = 0.014) (Figure 6). The height of scleral indentation in lattice lesions was positively correlated with the patient’s age (r = 0.51, *p* = 0.03) and negatively correlated with choroidal thickness within the lesion (r = −0.47, *p* = 0.02). 

## 4. Discussion

In this study, we found a high prevalence of scleral involvement in LD lesions, which included scleral indentation oriented along the greatest lesion dimension associated with choroidal thinning. This scleral involvement seems to be a specific finding for LD lesions and is either absent or very subtle in snail-track degeneration. Scleral involvement is almost undetectable in horseshoe retinal breaks. Among LD lesions, scleral indentation has a negative association with the presence of retinal breaks and local retinal detachment and may indicate a protective mechanism against rhegmatogenous retinal detachment. 

The mechanism of scleral involvement is not understood, but it is unlikely to represent primary changes leading to choroidal and retinal involvement since scleral changes have not been observed without degenerative retinal changes. Considering scleral protrusion as a result of other processes, we can suggest the role of vitreous tractions as its cause. Alternatively, structural changes in the sclera and its local contraction may follow degenerative choroidal and retinal changes. The second explanation is less relevant since the mechanism leading to degenerative changes in the retina is not well-understood. At the same time, the presence of vitreous adhesions and tractions in LD lesions is well-known, although the strength of the traction cannot be determined [9]. We, however, may suggest that tractional force is applied not only to the retina but to all tissue complex, including the choroid and sclera.

From this point of view, the height of the indentation may reflect both the strength of the traction and the binding of the retina, choroid, and sclera. In other words, if the retina, choroid, and sclera are tightly bound, the traction force is transmitted to the sclera and pulls it inward. In such cases, the magnitude of scleral indentation indicates the degree of tissue integrity, which prevents the occurrence of the retinal break. Moreover, scleral indentation weakens the vitreous tractions and may decrease the risk of retinal breaks. This explanation agrees with the decreased prevalence of retinal breaks and local retinal detachments in LD lesions with notable scleral indentation. 

Vitreous tractions play a crucial role in rhegmatogenous retinal detachment as the cause of retinal breaks. Moreover, tractions on the retinal flap also support the propulsion of liquified vitreous through the tear during eye movement [10]. Existing methods of managing rhegmatogenous retinal detachment are aimed at releasing or weakening causative vitreous tractions. Specifically, in PPV, the tractions are eliminated with the removal of the entire vitreous, while in scleral buckling, the indentation of the sclera weakens the vitreous traction. Additionally, the indentation creates a counterforce for the free movement of subretinal fluid and directs it to the vitreous cavity through the retinal break. These mechanisms, which drive the efficacy of scleral buckling, may also support the role of scleral indentation in LD as a protective mechanism. Although scleral indentation is a common finding in LD, we found an inverse association between the height of indentation and the presence of a full-thickness defect in LD lesions. Indeed, without indentation, tractions have greater force and are more likely to cause a retinal break. Similarly, no scleral indentations have been observed in horseshoe breaks. On the one hand, this indicates the high risk of retinal detachment from these breaks, but alternatively, it suggests the different nature of these breaks compared to those from LD lesions.

The correlation between the height of the indentation and the age of the patient may indicate that scleral indentation requires a substantial amount of time to develop and is likely to grow over time. The existence of the lesion over a long period potentially corresponds to a lower risk of rhegmatogenous retinal detachment, also leading to the conclusion that the higher the indentation, the lower the risk. From this point of view, the absence of indentation in high-risk horseshoe breaks suggests that these breaks result from new tractions before scleral involvement, which agrees with the need to treat symptomatic lesions only.

We believe that local scleral indentation in lattice lesions may result from deformation or local thickening of the sclera. However, the exact nature of this phenomenon remains unclear since the RPE layer and pigmentation of the lesion block OCT imaging of the sclera. Scleral deformation is a well-known phenomenon in highly myopic eyes, where it leads to the dome-shaped appearance of the macula [11]. Although this dome-shaped structure appears on the edge of the staphyloma and does not represent a local indentation but a part of global deformation, a number of studies have shown local scleral thickening in the dome-shaped macula [12]. The focal scleral nodule is another phenomenon associated with deformation of the inner scleral profile known to result from scleral thickening [13]. 

One theory relating to dome-shaped changes in the macula suggests choroidal thinning, also observed in lattice lesions, as the cause of scleral thickening. Although the relationship between choroidal thinning and scleral indentation is not clear, choroidal thinning may mask choroidal indentation to some degree. On the other hand, compression by scleral indentation could be a cause of choroidal thinning, leading to secondary alteration of the retina. However, neither in dome-shaped macular nor in focal scleral nodules do we see degenerative retinal changes as in lattice lesions [11,12,13]. This suggests the secondary or concomitant nature of scleral involvement in LD. From this point of view, the supposition that LD lesions result from abnormal vitreous adhesion looks relevant. Moreover, based on the difference in scleral and choroidal changes between lattice lesions and horseshoe retinal breaks, we suggest that lattice lesions in general may be an indicator for vitreous tractions with a reduced risk of retinal break formation. In other words, it can be referred to as a “mistake of survivor” and reflects notable protective changes in response to chronic vitreous tractions in contrast to acute new tractions causing horseshoe retinal breaks. At the same time, this does not exclude the complete formation of a retinal break in a particular lattice lesion.

Interestingly, previous studies that examined LD with OCT as well as histopathological studies did not demonstrate scleral involvement within lesions. We suggest this can be explained by a very specific orientation of the indentation along the lesion, which makes it indistinguishable on scans or sections passing through the lesion in other dimensions. The opportunity to display the lesion is also dependent on the characteristics of the OCT device, which is why early generation devices were limited in capturing peripheral lesions with neighboring retinal regions, which is necessary to identify the indentation. 

This study has several limitations. Firstly, only lesions successfully captured with OCT were analyzed, which excludes many peripheral lesions. Therefore, we cannot exclude the possibility that our findings may require clarification in terms of far-periphery lesions. Secondly, measurements of choroidal thickness and scleral indentation on the tilted scans are not as precise as those on horizontal scans. However, the tilt of the scan when displaying peripheral retina is unavoidable with the current concept of OCT. Thirdly, axial length, as a factor related to the vitreous tractions, may contribute to the height of scleral indentations in some lesions. However, axial length was not evaluated in our study, and its role requires further investigation. Finally, to confirm that scleral indentation has a protective effect, it would be useful to study the actual rate of retinal detachment from lesions with different indentation heights over time. 

Assessment of the risk of retinal detachment associated with lattice lesions is a subject of clinical interest, where OCT represents a relatively novel informative tool [4,8,14,15]. Based on the findings of this study, we propose scleral indentation as an anti-risk characteristic of lattice lesions. Moreover, the high prevalence of this feature in LD agrees with the current concept of laser prophylaxis, which is not required in the majority of asymptomatic cases.

Although snail-track degeneration has similar characteristics, including size, shape, orientation, and location, as LD, it carries only a small risk of retinal detachment. Our study revealed choroidal thinning below these lesions as in lattice lesions, while scleral involvement in snail-track degeneration is very limited or absent in the majority of cases. All of this therefore allows us to suggest that snail-tack degeneration can be a very mild or early type of LD. This is also supported by younger age and a lower myopia level in patients with snail-track lesions.

## 5. Conclusions

In conclusion, lattice degeneration lesions are associated with scleral involvement, which appears as scleral indentation with local choroidal thinning. This scleral indentation is oriented along the greatest lesion dimension, and its maximum height is negatively correlated with the presence of high-risk features, including retinal breaks and local retinal detachment. The height of the indentation seems to be an age-dependent function, possibly resulting from long-standing vitreous tractions. In contrast to lattice lesions, symptomatic horseshoe retinal breaks demonstrate no scleral involvement, suggesting that they develop from new and more dangerous vitreous tractions.

## Figures and Tables

**Table 1:** Characteristics of study groups.

	Lattice Degeneration	Snail-Track Degeneration	Horseshoe Break
Eyes, n	55	9	11
Males/females	27/28	5/4	3/8
Age, y	47.5 ± 15.8	31.3 ± 7.5 *	62.8 ± 10.7 †
Scleral indentation across the lesion, µm	227.2 ± 111.3	36.9 ± 51.2 *	22.0 ± 49.2 *
Scleral indentation along the lesion, µm	7.0 ± 23.8	0.0	0.0
Percentage choroidal thickness, %	49.0 ± 24.6	54.3 ± 28.9	99.6 ± 0.03 *†
Choroidal thickness within the lesion, µm	63.5 ± 38.4	45.1 ± 16.1	138.5 ± 18.4 *
Choroidal thickness outside the lesion, µm	135.6 ± 44.5	117.9 ± 26.7	140.6 ± 14.5

*—*p* < 0.05 compared to the lattice degeneration group; †—*p* < 0.05 compared to the snail-track degeneration group.

## Data Availability

The data presented in this study are available on request from the corresponding author.

## References

[1] Byer N.E. (1979). Lattice degeneration of the retina. Surv. Ophthalmol..

[2] Byer N.E. (1974). Changes in and prognosis of lattice degeneration of the retina. Trans. Am. Acad. Ophthalmol. Otolaryngol..

[3] Celorio J.M., Pruett R.C. (1991). Prevalence of lattice degeneration and its relation to axial length in severe myopia. Am. J. Ophthalmol..

[4] Cheung R., Ly A., Katalinic P., Coroneo M.T., Chang A., Kalloniatis M., Nivison-Smith L. (2022). Visualisation of peripheral retinal degenerations and anomalies with ocular imaging. Semin. Ophthalmol..

[5] Haimann M.H., Burton T.C., Brown C.K. (1982). Epidemiology of retinal detachment. Arch. Ophthalmol..

[6] Laatikainen L., Tolppanen E.M., Harju H. (1985). Epidemiology of rhegmatogenous retinal detachment in a Finnish population. Acta Ophthalmol..

[7] Maltsev D.S., Kulikov A.N., Burnasheva M.A., Chhablani J. (2021). Retro-mode scanning laser ophthalmoscopy in evaluation of peripheral retinal lesions. Graefes Arch. Clin. Exp. Ophthalmol..

[8] Maltsev D.S., Kulikov A.N., Shaimova V.A., Burnasheva M.A., Vasiliev A.S. (2023). Spotlight on Lattice Degeneration Imaging Techniques. Clin. Ophthalmol..

[9] Straatsma B.R., Zeegen P.D., Foos R.Y., Feman S.S., Shabo A.L. (1974). Lattice Degeneration of the Retina. Am. J. Ophthalmol..

[10] Machemer R. (1984). The Importance of Fluid Absorption, Traction, Intraocular Currents, and Chorioretinal Scars in the Therapy of Rhegmatogenous Retinal Detachments. Am. J. Ophthalmol..

[11] Gaucher D., Erginay A., Lecleire-Collet A., Haouchine B., Puech M., Cohen S.Y., Gaudric A. (2008). Dome-Shaped Macula in Eyes with Myopic Posterior Staphyloma. Am. J. Ophthalmol..

[12] Imamura Y., Iida T., Maruko I., Zweifel S.A., Spaide R.F. (2011). Enhanced Depth Imaging Optical Coherence Tomography of the Sclera in Dome-Shaped Macula. Am. J. Ophthalmol..

[13] Fung A.T., Waldstein S.M., Gal-Or O., Pellegrini M., Preziosa C., Shields J.A., Shields C.L. (2020). Focal Scleral Nodule. Ophthalmology.

[14] Shaimova V.A. (2017). Peripheral Retinal Degenerations: Optical Coherence Tomography and Retinal Laser Coagulation.

[15] Chu R.L., Pannullo N.A., Adam C.R., Rafieetary M.R., Sigler E.J. (2019). Morphology of Peripheral Vitreoretinal Interface Abnormalities Imaged with Spectral Domain Optical Coherence Tomography. J. Ophthalmol..

